# Mutagenic Analysis of the HIV Restriction Factor Shiftless

**DOI:** 10.3390/v14071454

**Published:** 2022-06-30

**Authors:** Niklas Jäger, Shreya Ahana Ayyub, Natalia Korniy, Frank Peske, Markus Hoffmann, Marina V. Rodnina, Stefan Pöhlmann

**Affiliations:** 1 Infection Biology Unit, German Primate Center, 37077 Göttingen, Germany; njaeger@dpz.eu (N.J.); mhoffmann@dpz.eu (M.H.); 2 Faculty of Biology and Psychology, Georg-August-University Göttingen, 37073 Göttingen, Germany; 3 Max Planck Institute for Multidisciplinary Sciences, 37077 Göttingen, Germany; shreya.ayyub@mpinat.mpg.de (S.A.A.); natalia.korniy@boehringer-ingelheim.com (N.K.); frank.peske@mpinat.mpg.de (F.P.); rodnina@mpinat.mpg.de (M.V.R.)

**Keywords:** –1 programmed ribosomal frameshifting, shiftless, C19orf66, restriction factor, HIV-1

## Abstract

The interferon-induced host cell protein shiftless (SFL) was reported to inhibit human immunodeficiency virus (HIV) infection by blocking the –1 programmed ribosomal frameshifting (–1PRF) required for expression of the Gag-Pol polyprotein. However, it is not clear how SFL inhibits –1PRF. To address this question, we focused on a 36 amino acids comprising region (termed required for antiviral activity (RAA)) that is essential for suppression of –1PRF and HIV infection and is missing from SFL short (SFLS), a splice variant of SFL with unknown function. Here, we confirm that SFL, but not SFLS, inhibits HIV –1PRF and show that inhibition is cell-type-independent. Mutagenic and biochemical analyses demonstrated that the RAA region is required for SFL self-interactions and confirmed that it is necessary for ribosome association and binding to the HIV RNA. Analysis of SFL mutants with six consecutive amino-acids-comprising deletions in the RAA region suggests effects on binding to the HIV RNA, complete inhibition of –1PRF, inhibition of Gag-Pol expression, and antiviral activity. In contrast, these amino acids did not affect SFL expression and were partially dispensable for SFL self-interactions and binding to the ribosome. Collectively, our results support the notion that SFL binds to the ribosome and the HIV RNA in order to block –1PRF and HIV infection, and suggest that the multimerization of SFL may be functionally important.

## 1. Introduction

The interferon (IFN) system constitutes an important innate defense against viral infection. Detection of viral pathogens by sensors of the IFN system induces expression of IFN, and binding of IFN to uninfected cells triggers signaling cascades that commandeer cells to transit into an antiviral state. The antiviral state is characterized by the IFN-induced expression of roughly 400 genes, many of which encode proteins that restrict virus infection (termed restriction factors) [1]. Several factors have been identified that restrict human immunodeficiency virus (HIV) infection, including APOBEC-3G, which blocks reverse transcription [2], and tetherin, which inhibits release of progeny particles from infected cells [3]. However, the list of antiviral factors known to contribute to IFN-induced inhibition of HIV infection is likely incomplete, and characterization of novel restriction factors might provide interesting insights into HIV pathogenesis and instruct novel antiviral approaches.

A new potential HIV restriction factor has been identified by Wang and colleagues who showed that the IFN-induced host cell protein shiftless (SFL), also known as C19orf66, blocks –1 programmed ribosomal frameshifting (–1PRF) in HIV-1 [4]. The –1PRF refers to ribosome backtracking by one nucleotide into an overlapping open reading frame (ORF), thereby generating a fusion protein encoded by upstream and downstream ORFs [5,6,7,8,9]. Such a strategy is used by many viruses to increase the coding capacity of their genomes and to regulate relative expression of viral proteins [8,9,10,11,12]. In the context of HIV infection, –1PRF is essential for the expression of the Gag-Pol polyprotein [13] and for viral infectivity and constitutes a target for SFL and potentially other restriction factors [4]. Indeed, the cellular proteins ELAVL1, HNRPH1, and HNRPH2, along with SFL, have been identified as inhibitors of –1PRF required for severe acute respiratory syndrome coronavirus 2 (SARS-CoV-2) gene expression [14,15], confirming that –1PRF is a target for cellular antiviral defenses. Apart from blocking HIV and SARS-CoV-2 infection, expression of SFL was shown to inhibit dengue virus (DENV) [16], Zika virus (ZIKV) [17,18], and hepatitis C virus (HCV) [19] infection. SFL can block –1PRF induced by a variety of viral sequences [4,20]. However, there is, at present, little evidence that –1PRF occurs and/or is required for ZIKV [11], DENV [21], and HCV [22] infection, suggesting that SFL might inhibit these viruses via other mechanisms. For instance, it is known that SFL induces lysosomal degradation of the ZIKV NS3 protein [17] and blocks HCV RNA replication by inhibiting the virus-induced increase of phosphatidylinositol-4-phosphate and by modulating the morphology of the cellular site of viral replication [19].

During HIV infection, SFL interacts with the translating ribosome and the –1PRF-inducing sequence of the viral RNA and causes premature translation termination at the site of frameshifting [4]. The mechanism underlying inhibition of –1PRF by SFL is incompletely understood. SFL is a 33 kDa protein that consists of 291 amino acids predicted to adopt a mixed α-β-fold. Sequence analysis identified a potential nuclear localization signal (NLS, 121–137), a nuclear export signal (NES, 261–269), a zinc-ribbon region (112–135), and a coiled-coil motif (261–286) [16]. Analysis of shiftless short (SFLS), a splice variant of SFL that lacks a 36 amino acid region (amino acids 164–199), revealed that SFLS does not bind the HIV –1PRF-inducing sequence and does not inhibit –1PRF and HIV infection [4]. Why the 36 amino acids comprising region, which we subsequently refer to as required for antiviral activity (RAA), is essential for suppression of –1PRF and HIV infection is, at present, unknown. However, the comparative analysis of SFL and SFLS might provide insights into how SFL blocks HIV infection.

Here, we show that SFL inhibits –1PRF in the context of HIV infection and that inhibition is cell-type-independent. We confirm that the RAA region is required for SFL association with the ribosome, SFL binding to the –1PRF-inducing sequence, suppression of –1PRF, and blockade of HIV infection. Furthermore, we show that the RAA region is required for formation of SFL oligomers and provide evidence that oligomerization and ribosomal association might be linked. Finally, we report RAA mutants that lost RNA binding and antiviral activity but were still capable of self-interactions and ribosome association and might thus be useful tools for further analyses of SFL antiviral activity.

## 2. Materials and Methods

### 2.1. Cell Culture 

HEK293T (human kidney cells, DSMZ no. ACC 635, DSMZ-German Collection of Microorganisms and Cell Cultures GmbH, Braunschweig, Germany), A549 (human epithelial lung cells, ATCC Cat# CRM-CCL-185; kindly provided by Georg Herrler, Institute of Virology University of Veterinary Medicine, Hannover, Germany) and TZM-bl (ARP5011, NIH AIDS Reagent Program, USA) cells were cultivated in Dulbecco’s Modified Eagle Medium (PAN-Biotech, Aidenbach, Germany) supplemented with 10% fetal bovine serum (FBS; Biochrom, Cambridge, UK) and penicillin–streptomycin (PAN-Biotech) at final concentrations of 100 U/mL (penicillin) and 0.1 mg/mL (streptomycin). All cell lines were incubated at 37 °C and 5% CO_2_ in a humidified atmosphere. For subcultivation and seeding, cells were either washed with phosphate-buffered saline (PBS) and detached with trypsin/EDTA (A549, TZM-bl; PAN-Biotech), or mechanically detached by resuspending the cells in fresh culture medium (HEK293T, HEK293). Next, the cell number was determined using the Neubauer chamber and cell suspensions were adjusted to 1.4 × 10^5^ cells/mL. Depending on the experimental setup, 2 mL (6 well-plate, 2.8 × 10^5^ cells/well), 1 mL (12-well-plate, 1.4 × 10^5^ cells/well), 250 µL (48 well-plate, 3.5 × 10^4^ cells/well), or 100 µL (96 well-plate, 1.4 × 10^4^ cells/well) of the cell suspension were seeded. Transfection of plasmids was either carried out by calcium–phosphate precipitation or by using Lipofectamine 2000 (Thermo Fisher Scientific, Waltham, MA, USA) according to the manufacturer’s instructions. Further, cell lines were routinely tested for contamination by mycoplasma.

### 2.2. Plasmids

For cloning of SFL (C19orf66, NCBI Reference Sequence: NP_060851.2) containing either a C-terminal cMYC or HA antigenic tag, total RNA was isolated from A549 cells using the RNeasy Mini Kit (Qiagen, Venlo, The Netherlands) and reverse-transcribed into cDNA employing the SuperScript^TM^ III First-Strand Synthesis System (Thermo Fisher Scientific, Waltham, MA, USA) as described in the manufacturer’s instructions for oligo-dTs. The coding sequence of SFL was PCR-amplified using appropriate forward primer in combination with reverse primer including C-terminal MYC or HA sequence (sequences available upon request). The amplified sequences were inserted into the pQCXIP vector [23] using NotI and PacI restriction sites. Restriction enzymes were purchased from New England Biolabs (Ipswich, MA, USA). For generation of expression plasmids for SFLS (NCBI Reference Sequence: NP_001295206.1) or the SFL mutants under study, we employed overlap-extension PCR using overlapping primers that introduce deletions into the RAA region of SFL open reading frame (sequences available upon request) and pQCXIP-SFL-HA-tag or pQCXIP-SFL-cMYC-tag as template. All PCR-amplified sequences were verified using a commercial sequencing service (Microsynth SeqLab, Göttingen, Germany).

To analyze –1PRF, a HIV-1 fragment (nt 1623–1694 NCBI Reference Sequence NC_001802.1) was cloned into a psiCHECK^TM^-2 (Promega, Madison, WI, USA) expression vector, which was used for the dual-luciferase assay (dual-luc-assay). The HIV-1 fragment contains a frameshifting site comprised of a heptanucleotide slippery site (SS) followed by a downstream stem-loop (SL) element, which are known to stimulate –1PRF in HIV-1 [13,24]. To allow translation initiation, an ATG start codon was introduced before the HIV-1 fragment. The HIV-1 fragment was inserted between the Renilla luciferase (RLuc) reporter (at the N-terminus) and the Firefly luciferase (FLuc) reporter (at the C-terminus). To avoid the signal disturbance due to possible reporter fusion, the RLuc–FLuc dimer is cleaved during translation by a P2A self-cleavage sequence (GGCGCCACCAACTTCTCCCTGCTGAAGCAGGCCGGCGACGTGGAGGAGAACCCCGGCCCC), which was inserted between the upstream RLuc reporter and the HIV-1 fragment. In addition, three stop codons (TAATAATAA) were inserted in 0 reading frame downstream of the HIV-1 fragment to prevent the translation of 0 frame beyond the HIV-1 sequence. Finally, the FLuc sequence was cloned into –1 reading frame by adding two guanine nucleotides ahead of FLuc to ensure that FLuc expression occurs only in case of –1PRF.

In the positive control (psiCHECK-PosCTR), the heptanucleotide slippery sequence was mutated in multiple positions (T_1_TTT_4_TTA_7_ to c_1_TTc_4_cTc_7_) to prevent –1PRF while preserving the wild-type amino acid sequence encoded by the slippery sequence. Furthermore, an additional cytosine nucleotide was inserted after the slippery sequence, which changes the open reading frame, allowing the synthesis of Fluc encoded by the –1 open reading frame. Thus, this control allows measuring the maximal signal of FLuc in the absence of –1PRF. In addition, a negative control (psiCHECK-NegCTR) was generated that contained a mutated slippery sequence and an additional cytosine nucleotide after the slippery sequence, as described above for the positive control. However, this vector lacks two guanine nucleotides before the FLuc sequence, thus placing FLuc into +1/−2 reading frame with regard to RLuc. This control allows to measure the background level of FLuc signal in the absence of –1PRF. Additional information regarding primer sequences and PCR procedure is available upon request. Finally, all PCR-generated sequences were verified using a commercial sequencing service (Microsynth SeqLab, Göttingen, Germany).

### 2.3. Analysis of Relative –1PRF Efficiency by Dual-Luciferase Assay

To analyze relative –1PRF efficiency, the cell lines tested were seeded in 48-well plates. The following day, cells were co-transfected with 250 ng/well of –1PRF reporter plasmid psiCHECK-HIV-1 jointly with 250 ng of the pQCXIP expression vector carrying the gene of interest (SFL, SFLS, SFL mutants). To detect potential unspecific effects of SFL, SFLS, and SFL mutants on translation, cells were co-transfected with the positive control psiCHECK-posCTR, which harbors a mutated slippery sequence placing RLuc and FLuc in the same frame, and with pQCXIP expression vectors harboring SFL, SFLS, and SFL mutants, as described above. A pQCXIP vector expressing GFP instead of SFL served as additional control. Transfection was carried out via Lipofectamine 2000 (Thermo Fisher Scientific, Waltham, MA, USA) following the manufacturer’s instructions. At 16 h post-transfection, the culture medium was replaced by fresh medium, and the cells were incubated for an additional 32 h. At 48 h post-transfection, culture medium was removed, and cells were lysed with 200 µL cell culture reagent (Promega, Madison, WI, USA) for 30–60 min at RT. For detection of the RLuc (0 reading frame) activity, 40 µL of each cell lysate was transferred into a white opaque-walled 96-well plate and 40 µL of the in-house-made RLuc substrate (Carl Roth, Karlsruhe, Germany) (dissolved in 137 mM NaCl, 2.7 mM KCl, 10 mM Na_2_HPO_4_, 1.8 M KH_3_PO_4_) was added. For detection of FLuc (–1 reading frame), FLuc substrate (PJK) was added instead of the RLuc substrate. Luminescence signals were quantified using a plate luminometer (Hidex, Turku, Finland). The background signal (i.e., signal measured for GFP transfected cells) was subtracted, and FLuc signals were normalized against the corresponding RLuc signals. Additionally, the RLuc/FLuc ratio obtained for the –1PRF reporter was normalized against the corresponding RLuc/FLuc ratio obtained for the positive control. Finally, the ratio obtained upon co-expression of GFP was set as 100%. One-way analysis of variance (ANOVA) with Dunnett’s multiple comparison test analysis was performed to test statistical significance (*, *p* ≤ 0.05; **, *p* ≤ 0.01; ***, *p* ≤ 0.001; *n* = 3). Paired Student’s *t*-test was performed to test statistical significance between SFL and SFLS (*, *p* ≤ 0.05; **, *p* ≤ 0.01; ***, *p* ≤ 0.001; HEK293, *n* = 3; A549, *n* = 10; TZM-bl, *n*= 8).

### 2.4. Analysis of Total Gag-Pol Expression by SDS-PAGE and Immunoblot

HEK293T cells were seeded in 12-well plates and co-transfected with an expression vector coding for HIV-1 Gag-Pol and pQCXIP vectors encoding SFL, SFLS, or SFL mutants, whereas an empty expression vector served as negative control. Transfection was carried out by calcium–phosphate precipitation. At 16 h post-transfection, the culture medium was replaced by fresh medium, and the cells were incubated for an additional 32 h. Subsequently, the cells were washed and then lysed with 250 µL of 2× SDS-sample buffer (0.03 M Tris-HCl, 10% glycerol, 2% SDS, 0.2% bromophenol blue, 1 mM EDTA) containing 10% 2-mercaptoethanol for 10 min at RT, followed by incubation at 96 °C for additional 10 min. Proteins in whole cell lysate (WCL) were separated via SDS-PAGE using 12.5% polyacrylamide gels and transferred onto a nitrocellulose membrane (0.2 µm pore size, GE Healthcare, Solingen, Germany). After blotting, the membranes were washed three times with PBS containing 0.1% Tween 20 (PBS-T) and were further blocked in 5% milk powder solved in PBS-T for 1 h at RT. The generated recombinant proteins SFL, SFLS, and SFL mutants were equipped with a cMYC-epitope, which was detected by using undiluted supernatant of hybridoma cells secreting a mouse anti-cMYC antibody (9E10). Membranes were incubated with primary antibodies overnight at 4 °C. Bound primary antibody was detected by incubation with an anti-mouse or anti-rabbit, horseradish peroxidase (HRP)-coupled secondary antibody (Dianova, Hamburg, Germany) at a dilution of 1:5000 for 1 h at RT. Visualization of bound secondary antibodies was achieved by using a chemiluminescence (ECL) solution (0.1 M Tris-HCl, pH 8.6, 250 µg/mL luminol, 1 mg/mL para-hydroxycoumaric acid, 0.3% H_2_O_2_) in combination with the ChemoCam imaging system and the ChemoStarProfessional software (version v.0.3.23, INTAS, Göttingen, Germany). After imaging the recombinant proteins, the membranes were stripped of bound antibodies by incubation with stripping buffer (Carl Roth, Karlsruhe, Germany) according to the manufacturer’s instructions and p160 Gag-Pol, p55 Gag, and p24 Capsid was detected using undiluted supernatant of hybridoma cells secreting a mouse anti p24 antibody (1183 HT12 5C). For the loading controls, membranes were stripped again and β-actin (ACTB) or uL3 expression was detected by using anti-β-actin (A2066, Sigma-Aldrich, St. Louis, MO, USA) or anti-uL3 (Atlas antibodies, HPA003365) antibodies diluted 1:1000 and 1:500, respectively, in a 5% BSA/PBS-T solution. Band intensities were quantified using the ImageJ software (version 1.8.0, https://imagej.nih.gov/ij/).

### 2.5. Generation of HIV Reporter Particles and Transduction of Target Cells

293T cells were seeded in T25 flasks. The following day, cells were transfected with plasmids encoding (i) an HIV-derived vector harboring a Firefly luciferase reporter gene, (ii) HIV Gag-Pol for packaging of the HIV vector, and (iii) the glycoprotein of the vesicular stomatitis virus (VSV-G) together with plasmids encoding for SFL, SFLS, or SFL mutants. A vector expressing GFP instead of SFL served as a control. Calcium–phosphate precipitation was used for transfection. At 12 h post-transfection, medium was replaced with fresh medium. At day three post-transfection, supernatants of transfected 293T cells were collected and cleared from cellular debris by centrifugation at 5000 rpm for 10 min at room temperature. Subsequently, naïve 293T cells seeded in 96-well plates were transduced with the cleared supernatants (quadruplicates, 1:10 diluted). At 48 h post-transduction, culture medium was removed, and cells were lysed with 55 µL cell culture reagent (Promega, Madison, WI, USA) for 30–60 min at RT. For detection of the FLuc activity, 50 µL of each cell lysate was transferred into a white opaque-walled 96-well plate and 50 µL of FLuc substrate (PJK, Kleinbittersdorf, Germany) was added. Luminescence signals were quantified using a plate luminomenter (Hidex, Turku, Finland) and indicate transduction efficiency. Finally, background signal was subtracted and signals measured upon co-expression of GFP were set as 100%.

### 2.6. Analysis of SFL Interactions via Co-Immunoprecipitation

HEK293T cells were seeded in 6-well plates, as described above. The following day, cells were co-transfected (via Lipofectamine 2000) with 1 µg of expression plasmids coding for SFL, SFLS, or SFL mutants carrying a cMYC-tag together with either SFL, SFLS, or SFL mutants fused with an HA-tag at the C-terminus. A vector expressing GFP instead of the recombinant proteins served as control. At 48 h post-transfection, cells were washed once with cold PBS following lysis with 500 µL RIPA lysis buffer (50 mM Tris, pH 8.0, 150 mM NaCl, 1.0% Triton X-100, 0.5% sodium deoxycholate, 0.1% SDS) containing protease inhibitor cocktail cOmplete^TM^ (Roche, Basel, Switzerland) by incubation for 60 min on ice. Subsequently, cell lysates were collected in 1.5 mL tubes and centrifuged for 15 min at 12,700 rpm at 4 °C, followed by mixing 200 µL of the supernatant with 50 µL of protein A-Sepharose (1 g protein A-Sepharose in 10 mL H_2_O, Sigma-Aldrich, St. Louis, MO, USA) containing 1 µL of anti-HA antibody (rabbit, H6908, Sigma-Aldrich, St. Louis, MO, USA). Additionally, 50 µL of the supernatant of the whole cell lysate (WCL) were mixed with 50 µL 2 × SDS-sample buffer and were stored overnight at 4 °C. The Protein A-Sepharose-lysate mixture was incubated overnight at 4 °C in an overhead shaker. The next day, Protein A-Sepharose samples were centrifuged for 5 min at maximum speed at 4 °C and supernatant was removed. Subsequently, samples were washed carefully with 500 µL of cold RIPA lysis buffer (without protease inhibitors). This washing step was repeated three times. Finally, 60 µL of 2× SDS-sample buffer was added to the pelleted complexes and Protein A-Sepharose samples and WCL were further incubated for 15 min at 96 °C, before analysis via immunoblot, which was conducted as described above. For the detection of the proteins carrying an HA-epitope, an HA-specific mouse antibody (H3663, Sigma-Aldrich, St. Louis, MO, USA) was used at a dilution of 1:2000 in 5% BSA/TBS-T. Similarly, proteins carrying a cMYC-epitope were detected by a cMYC-specific mouse antibody (MA1-980, Thermo Fisher Scientific, Waltham, MA, USA). To avoid signal interference due to stripping procedure, HA- and cMYC-tagged proteins were detected on separate gels. As a loading control for WCL samples, ACTB was detected by a β-actin specific mouse antibody (1:2000, A5441, Sigma-Aldrich, St. Louis, MO, USA). Incubation and imaging procedure were carried out as described above.

Signal intensity of the protein bands were quantified and signals of the WCL samples were normalized against the ACTB signal obtained in WCLs. Protein of interest in the protein A-Sepharose samples was normalized against the HA-band.

### 2.7. Ribosome Pelleting Assay

HEK293T cells were seeded in 6-well plates as previously described. The following day, cells were transfected with 2 µg of expression plasmids coding for recombinant SFL, SFLS, or SFL mutants carrying a cMYC-tag at the C-terminus. A vector expressing dsRed tagged with a cMYC-epitope and an empty vector served as controls. At 48 h post-transfection, cells were harvested in cold medium and subsequently pelleted via centrifugation for 3 min at 2300 g and washed with ice-cold PBS. Cells were lysed with 250 µL RNC buffer (50 mM HEPES-KOH, pH 7.4, 100 mM KCl; 5 mM MgCl_2_) supplemented with 100 µg/mL cycloheximide and Triton X-100 at a final concentration of 0.1%. After a 2 h incubation on ice, lysates were centrifuged for 30 min at 13,400 g at 4 °C to remove cell debris. A total of 180 µL of the supernatant was loaded onto 800 µL of a 0.5 M sucrose cushion in RNC buffer and the remaining whole cell lysate served as input control. Samples were centrifuged for 1 h at 100,000 rpm at 4 °C (MLA-130 rotor, Beckman Coulter, Krefeld, Germany), and, subsequently, the supernatant was discarded. The ribosomal pellet was carefully washed with 100 µL RNC buffer, resuspended in 45 µL of RNC buffer, and proteins of interest were detected via immunoblotting.

### 2.8. mRNA Binding Assay

The HIV-1 mRNA used for the mRNA binding assay is a 428 nt transcript used in a previous study [25]. The sequence of the control *E. coli* lpp mRNA which lacks a FSE is as follows: 

5′GGGCUACAUGGAGAUUAACUCAAUCUAGAGGGUAUUAAUAAUGUCCAGCAACGCUAAAAUCGAUCAGCUGUCUUCUGACGUUCAGACUCUGAACGCUAAAGUUGACCAGCUGAGCAACGACGUGAACGCAAUGCGUUCCGACGUUCAGGCUGCUAAAGAUGACGCAGCUCGUGCUAACCAGCGUCUGGACAACAUGGCUACUAAAUACCGCAAGUAAUAGUACCUGUGAAGUGAAAAAUGGCGCACAUUGUGCGCCAUUUUUUU3′.

mRNAs were labeled with Atto488 using a method adapted from earlier studies [26,27,28]. Briefly, 5 nmol mRNA were oxidized at the 3′ ribose by treatment with 1 mM KIO_4_ and 0.1 M sodium acetate (pH 5.3) for 45 min on ice. Oxidation was stopped by incubation with 33 mM ethylene glycol for 10 min on ice. After two rounds of isopropanol precipitations from 0.15 M sodium acetate (pH 5.3), the mRNA was treated with 2 mM Atto488 hydrazide (ATTO-TEC GmbH, Siegen, Germany) in 0.1 M sodium acetate (pH 5.3) and incubated in the dark for 1 h at 37 °C. After one round of isopropanol precipitation and one round of ethanol precipitation, the mRNA was dissolved in 50 µL water and quantified by measuring absorbance at 500 nm.

HEK293T cells were seeded in 12-well plates and transfected with an expression vector coding for recombinant SFL, SFLS, or SFL mutant equipped with C-terminal MYC-tag as described above. A GFP expression vector, where GFP was expressed without a cMYC tag, served as the negative control. At 48 h post-transfection, cells were lysed with 450 µL RIPA buffer by incubation for 60 min on ice. Cell lysates were collected and centrifuged for 15 min at 12,700 rpm at 4 °C. Subsequently, 215 µL of clarified lysate was added to 25 µL Dynabeads (10002D, Thermo Fisher Scientific, Waltham, MA, USA) containing 1 µL anti-MYC antibody and mixed using a Rotator/Mixer (Intelli-Mixer RM-2M, Elmi, Riga, Latvia) for 1 h at 4 °C. Afterwards, 335 pmol of Atto488-labeled HIV-1 frameshift RNA or control mRNA (or 335 pmol of both as in Figure 7B) were added to the Dynabead mixture and samples were mixed by rotation for an additional 2 h at 4 °C. Samples were washed three times by using a magnetic stand with RNC buffer (600 mM NaCl). Dynabeads were incubated with 500 µL water containing 2 µg/mL proteinase K and 0.1% SDS for 30 min at 55 °C in a shaker operated at 1000 rpm. After adding 500 µL phenol/chloroform/isoamyl alcohol (Carl Roth, Karlsruhe, Germany), samples were vortexed for 30 s and centrifuged at 16,000× *g* for 2 min. mRNA was precipitated from 400 µL of supernatant with 1 mL 100% ethanol, 0.1 M Na acetate, and 35 µg/mL glycogen overnight at −20 °C, followed by centrifugation for 15 min at 16,000 g at 4 °C and washing with 500 µL 70% ethanol. The mRNA was resuspended in 10 µL water by shaking for 10 min at 65 °C. Finally, samples were loaded on a urea PAGE with formamide RNA loading dye [29], and Atto488-labeled mRNA was detected using the Amersham Typhoon scanner (Cytiva, Freiburg, Germany) with the Cy2 setting.

### 2.9. Expression and Purification of SFL

SFL was cloned by Gibson assembly [30] into a pET28b vector (a kind gift from Prof. Ralf Ficner, University of Göttingen, Germany) with an N-terminal GST-tag. SFL was expressed in *E. coli* Lemo21 cells (New England Biolabs, Ipswich, MA, USA) on induction with 1 mM IPTG overnight at 15 °C in LB medium supplemented with kanamycin (25 µg/mL). The cells were harvested and pellets were resuspended in 20 mM Tris-HCl, pH 7.5, 500 mM KCl, 2 mM DTT, 10% glycerol, inhibitor cocktail cOmplete^TM^ (Roche, Basel, Switzerland), lysozyme, and a trace of DNaseI. Cells were homogenized using a Dounce homogenizer followed by lysis using an Emulsiflex apparatus (Avestin, Ottawa, ON, Canada), and the extract was centrifuged in a Ti50.2 rotor at 35,000 rpm for 30 min at 4 °C. The supernatant was loaded on a GSTrap FF column (GE Healthcare, Solingen, Germany). The target protein was eluted with a buffer containing 20 mM Tris-HCl, pH 7.5, 500 mM KCl, 2 mM DTT, 10% glycerol, and 30 mM glutathione. The eluted protein was mixed with TEV protease and incubated overnight at 4 °C to cleave the GST-tag. The protein was further purified using a size-exclusion chromatography column HiLoad 26/60 Superdex 200 preparative grade equilibrated in 20 mM Tris-HCl, pH 7.5, 100 mM KCl, 2 mM DTT, and 10% glycerol (GE Healthcare, Solingen, Germany). The eluted protein was concentrated by Vivaspin 20, 10,000 MWCO centrifugal concentrator (Sartorius, Göttingen, Germany) and loaded on a HiTrapQ-HP anion exchange column for the final step of purification. The HiTrapQ-HP column was equilibrated in 20 mM Tris-HCl pH 7.5, 100 mM KCl, 2 mM DTT, and 10% glycerol, and elution was carried out with a continuous KCl gradient (100 mM to 1 M) using 20 mM Tris-HCl, pH 7.5, 1 M KCl, 2 mM DTT, and 10% glycerol. SFL was quantified from SDS PAGE relative to bands from the Perfect Protein Marker (Merck Millipore, Darmstadt, Germany) using MultiGauge software (version 2.3, Fujifilm, Tokyo, Japan).

### 2.10. Analytical Gel Filtration

Experiments were conducted using a Superdex 200 Increase 10/300 GL (GE Healthcare, Solingen, Germany) equilibrated in gel filtration buffer (20 mM Tris-HCl, pH 7.5, 100 mM KCl, 2 mM DTT, 10% glycerol). EF-G (200 pmol) was mixed with EF-Tu (4000 pmol) and loaded on the column and the elution profile was recorded to serve as controls for protein size. The column was washed with one column volume of gel filtration buffer and SFL (3840 pmol) was loaded on the column and the elution profile was recorded. Subsequently, the column was equilibrated with one column volume of gel filtration buffer containing 6 M urea. SFL (3840 pmol) was treated with 6 M urea and 10 mM 2-mercaptoethanol, incubated for 15 min at 37 °C, loaded on the column, and the elution profile was recorded. All the proteins were injected at a flow rate of 0.4 mL/min.

### 2.11. Statistical Analysis

One-way analysis of variance (ANOVA) with Dunnett’s multiple comparison post-test was used to test statistical significance to the control. Only *p* ≤ 0.05 was considered statistically significant (*p* > 0.05 (n.s., not significant), *p* ≤ 0.05 (*), *p* ≤ 0.01 (**), *p* ≤ 0.001 (***)). To compare two independent values, unpaired, two-tailed Student’s t-test was used (n.s., *p* > 0.05; **, *p* ≤ 0.05). For all statistical analyses, the GraphPad Prism 7 software package was used (version 7.03, GraphPad Software, San Diego, CA, USA).

## 3. Results

### 3.1. SFL Suppresses –1PRF of HIV in a Cell Line-Independent Fashion

**Figure 1:**
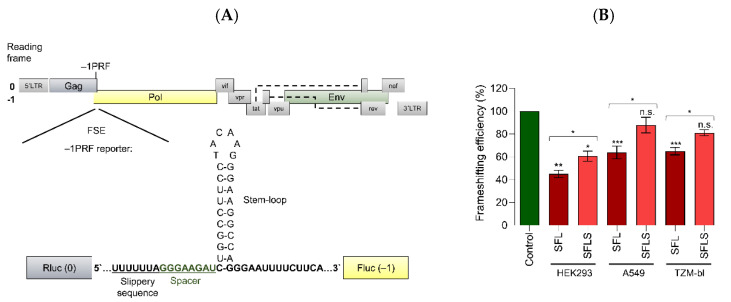
SFL inhibits HIV –1PRF in a cell-line-independent fashion. (**A**) Schematic illustration of the HIV-1 genome, including the site of –1PRF and the FSE. For quantification of –1PRF, the FSE sequence including the heptanucleotide slippery sequence, spacer, and stem-loop from the HIV-1 mRNA was inserted between Renilla luciferase (Rluc) (0 reading frame) and Firefly luciferase (Fluc) (–1 reading frame). (**B**) HEK293, A549, or TZM-bl cells were co-transfected with plasmids encoding the –1PRF reporter cassette and either SFL or SFLS. Co-transfection of a plasmid-encoding GFP served as a control. RLuc and FLuc signals were quantified at 48 h post-transfection. The FLuc signal was normalized against RLuc signal and signals measured upon co-transfection of the control plasmid were set as 100%. The average of three to ten biological replicates (HEK293, *n* = 3; A549, *n* = 10; TZM-bl, *n* = 8) carried out with technical triplicates is shown. Error bars indicate SEM. One-way analysis of variance (ANOVA) with Dunnett’s multiple comparison test was performed to determine whether differences between cells expressing GFP (control) and SFL or SFLS were statistically significant (not significant [n.s.]; *p* ≥ 0.05; *, *p* ≤ 0.05; **, *p* ≤ 0.01; ***, *p* ≤ 0.001). Unpaired Student’s *t*-test was performed to test whether differences between cells expressing SFL or SFLS were statistically significant (* *p* ≤ 0.05). SFL, shiftless; SFLS, shiftless short.

We first sought to confirm inhibition of HIV –1PRF by SFL, but not SFLS, and to determine whether inhibition is cell-type-dependent. For this, we employed a dual-luciferase assay based on the –1 frameshifting stimulation element (FSE) present in the HIV-1 isolate NL4-3 (Figure 1A). The HIV FSE comprises a so-called slippery sequence and a downstream stimulatory element, a stem-loop, or, in the context of HIV-1 group O viruses, a pseudoknot [5,13,31,32]. For the dual-luciferase assay, the FSE is flanked by a Renilla-luciferase (RLuc) open reading frame (ORF) located 5′ from the FSE and a Firefly-luciferase (FLuc) ORF 3′ of the FSE in the –1 reading frame. As a consequence, FLuc is only translated if –1PRF occurs, with the ratio between FLuc and RLuc activity indicating the efficiency of –1PRF (Figure 1A).

To determine whether SFL suppresses –1PRF in a cell-type-dependent fashion, we performed the dual-luciferase assay in the human embryonal kidney cell line HEK293, the human lung cell line A549, and the indicator cell line TZM-bl [33] derived from the human cervical carcinoma cell line HeLa. HEK293 cells are frequently used for biomedical research due to their high transfectability. A549 cells are commonly employed for analysis of viral infection of lung cells. TZM-bl cells express the reporter genes Firefly luciferase and β-galactosidase upon HIV infection and are a popular tool to quantify HIV infectivity [34]. To determine –1PRF and its suppression by SFL and SFLS, the cells were co-transfected with the above-described reporter plasmid and plasmids encoding SFL or SFLS. Transfection of a GFP-encoding plasmid served as negative control. In the absence of SFL, frameshifting efficiency (calculated as the ratio of FLuc/RLuc measured upon co-expression of GFP) was between 5% and 11% in all cell lines tested, as expected [35,36,37,38]. In order to allow convenient comparison between different cell lines, signals measured upon co-expression of GFP were set as 100% for each cell line. In HEK293 cells, SFL reduced frameshifting efficiency by ~56% while expression of SFLS diminished frameshifting by ~38% (Figure 1B). Suppression of –1PRF by SFLS was unexpected and might be due to high levels of protein expression in HEK293 cells. In A549 and TZM-bl cells, SFL reduced –1PRF by roughly 35%, while SFLS expression diminished –1PRF by 12 to 18% (Figure 1B), but the latter effect was not statistically significant. Thus, SFL suppresses –1PRF in a cell-line-independent fashion, although efficiency of inhibition can vary, while SFLS is largely, but not completely, inactive regarding suppression of –1PRF.

### 3.2. Deletions in the C-Terminal Portion of the RAA Region Abrogate –1PRF Inhibition

**Figure 2:**
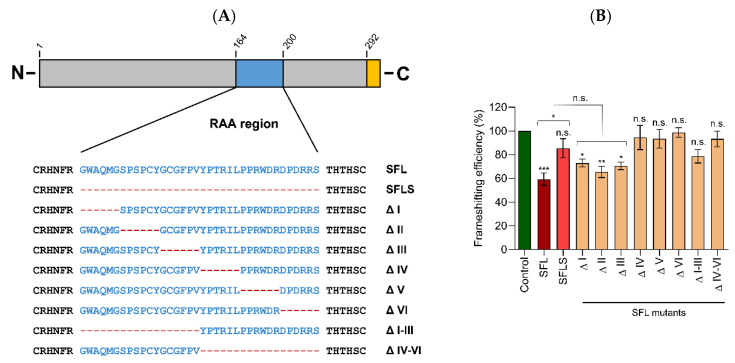
Deletions in the C-terminus of the RAA abrogate inhibition of –1PRF. (**A**) Sequences deleted from the RAA are shown. A C-terminal antigenic tag added for analysis of protein expression is shown in orange. (**B**) A549 cells were transfected with plasmids encoding SFL, SFLS, or SFL mutants together with –1PRF reporter vector. Luciferase signals in cell lysates were measured at 48 h post-transfection. A GFP expression plasmid served as a control. The average of five biological replicates conducted with technical triplicates is shown; error bars indicate SEM. One-way analysis of variance (ANOVA) with Dunnett‘s multiple comparison test was performed to determine whether differences between cells expressing GFP (control) and SFL, SFLS, or SFL mutants were statistically significant (not significant [n.s.]; *, *p* ≤ 0.05; **, *p* ≤ 0.01; ***, *p* ≤ 0.001; *n* = 5). Unpaired Student’s *t*-test was performed to test whether differences between cells expressing SFL or SFLS and cells expressing SFLS and mutants Δ I, Δ II, or Δ III were statistically significant (not significant [n.s.]; *, *p* ≤ 0.05; **, *p* ≤ 0.01; ***, *p* ≤ 0.001; *n* = 5). SFL, shiftless; SFLS, shiftless short.

We next asked which amino acids in the RAA (the SFL region that is absent from SFLS) are required for suppression of –1PRF. As the structure of the RAA region is not known, we could not rationally design mutations and have, therefore, chosen to sequentially delete blocks of six amino acids in this region (Figure 2A). In addition, we generated SFL mutants lacking the N- or C-terminal 18 acids of the RAA. To explore whether these mutations modulate SFL-mediated suppression of –1PRF uncoupled from Gag-Pol expression, we performed the dual-luciferase assay using A549 cells, in which the differential inhibitory activity of SFL, as compared to SFLS, was more pronounced than in HEK293 cells (Figure 1B). SFL mutants ΔI, ΔII, and ΔIII, which harbor deletions in the N-terminal part of the RAA, showed intermediate phenotypes between SFL and SFLS, whereas deletions within the C-terminal half (mutants ΔIV, ΔV, ΔVI) or deletion of the complete N-terminal (mutants ΔI–ΔIII) or C-terminal (mutants ΔIV–VI) portion of the RAA resulted in the loss of –1PRF inhibition (Figure 2B). Collectively, these results confirm that the RAA is required for suppression of –1PRF, in agreement with published data [4].

### 3.3. Deletions in the RAA Reduce the Ability of SFL to Suppress Gag-Pol Expression and HIV Infectivity

**Figure 3:**
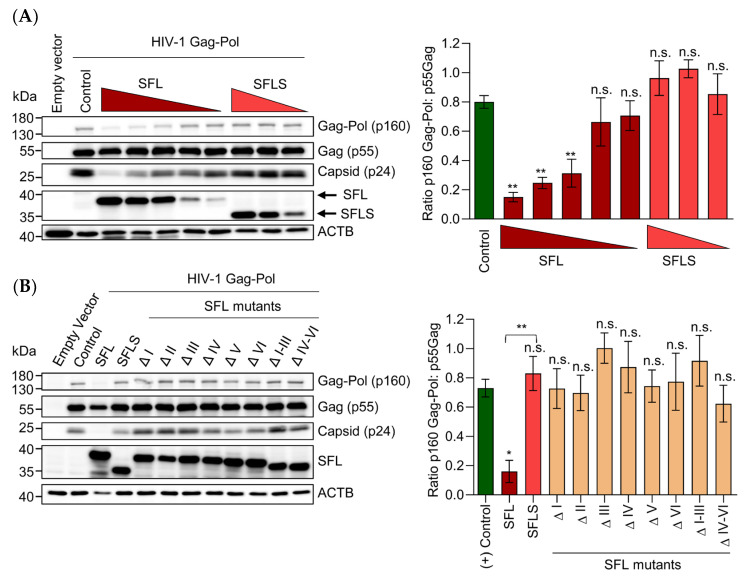
Deletions in the RAA are not compatible with suppression of Gag-Pol expression. HEK293T cells were co-transfected with a vector encoding HIV-1 Gag-Pol and (**A**) different amounts of plasmids encoding SFL (1 µg, 0.75 µg, 0.5 µg, 0.1 µg, 0.05 µg) or SFLS (1 µg, 0.75 µg, 0.5 µg) or (**B**) constant amounts of plasmids encoding SFL, SFLS, and the indicated SFL mutants. Co-transfection of the Gag-Pol encoding plasmid with a GFP expression plasmid served as control. Cells were harvested 48 h post-transfection and protein expression was analyzed by immunoblot. Expression of SFL, SFLS, and SFL mutants was analyzed with an antibody against the C-terminal MYC antigenic tag. Gag-Pol (p160), Gag (p55), and capsid (p24) expression was detected using an anti-p24 antibody. Expression of β-actin (ACTB) served as a loading control. Representative blots are shown in the left panel; irrelevant lanes were excised. Three biological replicates were quantified using the software ImageJ and the ratio of Gag-Pol and Gag expression normalized to ACTB was calculated. Signals measured for the control (co-transfection of GFP expression plasmid) were set as 100%. The averages of three replicates are shown in the right panel, and error bars indicate SEM. One-way analysis of variance (ANOVA) with Dunnett‘s multiple comparison test was performed to determine whether differences between cells expressing GFP (control) and SFL, SFLS, or SFL mutants were statistically significant (not significant [n.s.]; *, *p* ≤ 0.05; **, *p* ≤ 0.01; *n* = 3). Unpaired Student’s *t*-test was performed to test whether differences between cells expressing SFL or SFLS were statistically significant **, *p* ≤ 0.01, *n* = 3). SFL, shiftless; SFLS, shiftless short.

We next investigated whether the deletions in the RAA region interfere with suppression of Gag-Pol expression and HIV infection. For these experiments, we employed HEK293T cells where Gag-Pol expression was more reproducible than in A549 cells (not shown), potentially due to differences in transfection efficiency. To analyze suppression of Gag-Pol expression, the cells were co-transfected with plasmids encoding NL4-3 Gag-Pol and SFL, SFLS, or SFL deletion mutants. We found that SFL, but not SFLS, inhibited expression of Gag-Pol and p24 capsid (CA), a processing product of Gag-Pol, in a dose-dependent manner (Figure 3A). In contrast, the expression of p55 Gag remained unaffected (Figure 3A), confirming published data [4]. SFLS and the SFL deletion mutants failed to inhibit Gag-Pol expression (Figure 3B). The effects of the deletion mutants on p24 expression were generally consistent with those measured in the absence of SFL or in the presence of SFLS, confirming that any alteration of the RAA region abrogated interference with Gag-Pol expression (Figure 3B).

**Figure 4:**
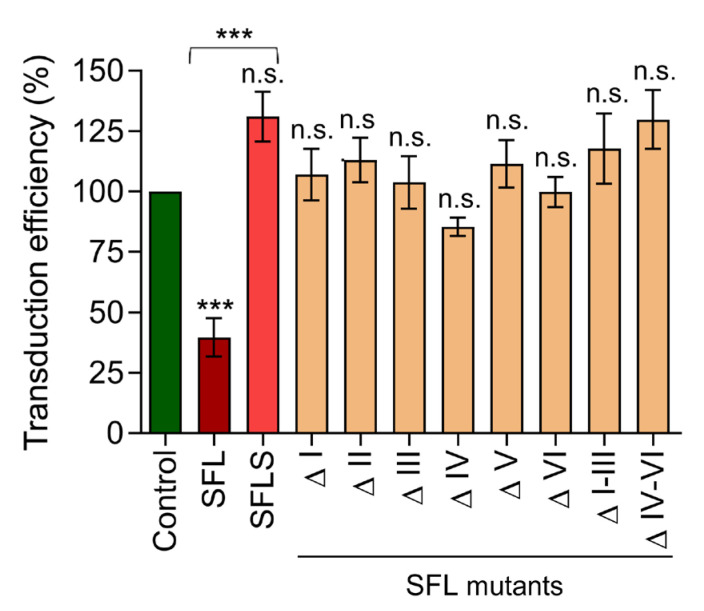
Deletions within the RAA abrogate SFL-mediated suppression of viral infectivity. HIV reporter particles containing a luciferase encoding vector genome and harboring the glycoprotein of vesicular stomatitis virus (VSV-G) were produced in the presence and absence of SFL, SFLS, or SFL mutants. Transfection of a GFP expression plasmid served as negative control. At 72 h post-transfection, supernatants were collected and equal volumes used for transduction of HEK293T cells. At 72 h post-transduction, the luciferase activity in cell lysates was measured. Transduction measured for control particles produced in the absence of SFL was set as 100%. The average of five biological replicates carried out with technical triplicates is shown; error bars indicate SEM. One-way analysis of variance (ANOVA) with Dunnett‘s multiple comparison test was performed to determine whether differences between cells expressing GFP (control) and SFL, SFLS, or SFL mutants were statistically significant (not significant [n.s.]; ***, *p* ≤ 0.001; *n* = 5). Unpaired Student’s *t*-test was performed to test whether differences between cells expressing SFL or SFLS were statistically significant (***, *p* ≤ 0.001; *n* = 5). SFL, shiftless; SFLS, shiftless short.

We next asked whether the failure of SFL mutants to inhibit –1PRF and Gag-Pol expression translated into loss of antiviral activity. To address this question, we generated HIV particles by co-expression of an HIV-derived vector genome harboring a luciferase reporter gene, the glycoprotein of the vesicular stomatitis virus (VSV-G) and HIV Gag-Pol in the presence and absence of SFL, SFLS, or SFL mutants and analyzed transduction efficiency. The expression of SFL in particle-expressing cells reduced transduction by ~60%, while neither SFLS nor the SFL mutants interfered with transduction efficiency (Figure 4), despite robust expression (Figure 3B). Collectively, these results suggest that any deletion in the RAA region is incompatible with efficient suppression of Gag-Pol expression and production of infectious particles.

### 3.4. Deletions in the RAA N-Terminus Reduce SFL Self-Interactions

**Figure 5:**
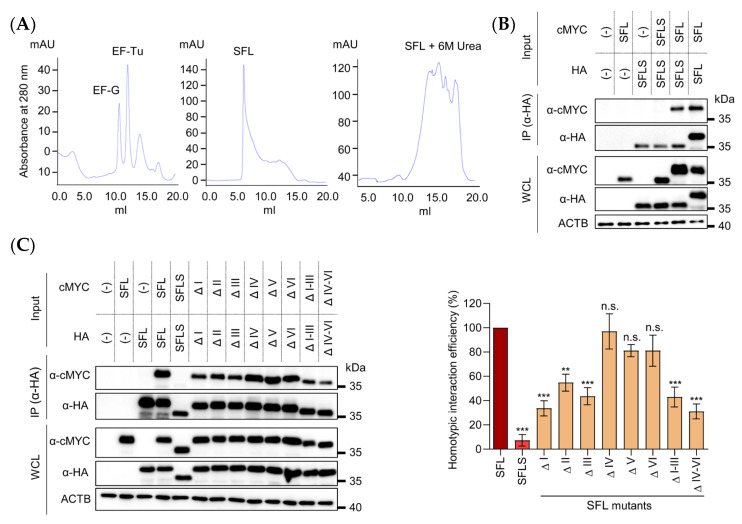
Effect of RAA deletions for SFL self-interactions. (**A**) Analytical gel filtration profiles for (i) EF-G and EF-Tu, (ii) SFL and (iii) SFL treated with 6 M urea. The indicated proteins were loaded on a Superdex 200 Increase 10/300 GL column and elution profiles were recorded. EF-G and EF-Tu served as controls for protein size. (**B**) HEK293T cells were co-transfected with plasmids encoding SFL or SFLS equipped with a C-terminal HA- or MYC-antigenic tag. At 48 h post-transfection, proteins carrying an HA-tag were immunoprecipitated (IP) and detected with an antibody against the HA antigenic tag. Co-precipitated proteins were detected with an antibody against the MYC antigenic tag. Transfection of empty vector or single transfection of plasmids expressing either cMYC-tagged SFLS or HA-tagged SFL served as negative controls. Expression of the proteins in whole cell lysates (WCL) served as control of input proteins. Expression of β-actin (ACTB) was determined as loading control. A representative immunoblot is shown from which irrelevant lanes were excised. Similar results were obtained in two separate experiments. (**C**) HEK293T cells were co-transfected with plasmids encoding SFL, SFLS, or SFL mutants carrying a C-terminal HA- or MYC-tag. Anti-HA antibody was used for immunoprecipitation, and precipitated proteins were detected with anti-HA antibody and co-precipitated proteins with anti-MYC antibody. Protein expression in WCL was checked by immunoblot. Expression of ACTB served as a loading control. A representative immunoblot is shown in the left panel; irrelevant lanes were excised. Similar results were obtained in two separate experiments. Three biological replicates were quantified using the software ImageJ and their average is shown in the right panel. cMYC signals in the WCL were normalized to ACTB signals (and are referred to as input). Further, cMYC signals in the IP reactions were normalized to the respective input signals. Finally, the ratio between cMYC signals in the IP and WCL was determined and the ratio obtained for SFL was set as 100%. Error bars indicate standard error of the mean (SEM). One-way analysis of variance (ANOVA) with Dunnett’s multiple comparison test was performed to determine whether differences between cells expressing SFL and SFLS or SFL mutants were statistically significant (not significant [n.s.]; **, *p* ≤ 0.01; ***, *p* ≤ 0.001; *n* = 3). SFL, shiftless; SFLS, shiftless short; WCL, whole cell lysate.

We next studied why deletions in the RAA region interfered with SFL antiviral activity. Previous studies suggested that SFL forms multimers and that multimerization might be required for inhibition of –1PRF [4]. Size exclusion chromatography experiments with recombinant SFL revealed that SFL (monomer size 33 kDa) eluted from the gel filtration column earlier than two larger control proteins, EF-G (77 kDa) and EF-Tu (43 kDa), suggesting that SFL forms oligomers. This effect could be reversed by treatment with 6 M urea, which should disrupt oligomerization and cause partial protein unfolding; the latter may explain the multiplicity of protein peaks observed in the presence of 6 M urea (Figure 5A, right panel). To further investigate whether SFL forms oligomers, we employed co-immunoprecipitation. SFL, SFLS, or SFL deletion mutants carrying HA- or MYC-tags were co-expressed in HEK293T cells, followed by immunoprecipitation with anti-HA antibody and detection of binding partners with anti-MYC antibody. We first examined whether homotypic SFL–SFL and SFLS–SFLS, as well as heterotypic SFL–SFLS, interactions could be detected by immunoprecipitation. SFL co-precipitated with SFL and SFLS, while SFLS did not co-precipitate SFLS, although both proteins were readily detectable in whole cell lysates (WCL) and were pulled down by the anti-HA antibody (Figure 5B,C, upper panel, anti-HA). We then analyzed homotypic interactions between SFL deletions mutants. All SFL deletion mutants were able to self-interact (Figure 5C), although with somewhat different efficiency. Deletions of blocks of six amino acids in the N-terminal portion and removal of the N- or C-terminal 18 amino acids of the RAA region reduced homotypic interactions by~40 to 60% (Figure 5C). In contrast, deletions of blocks of six amino acids in the C-terminal part had no appreciable effect (Figure 5C).

Collectively, our results suggest that binding of the RAA to a so-far unknown region of SFL, rather than RAA–RAA interactions, are responsible for SFL–SFL multimerization. It should be noted that deletions in the C-terminal portion of the RAA (mutants ΔIV, ΔV, ΔVI) had little impact on SFL–SFL interactions but fully abrogated antiviral activity (Figure 4). This may indicate that the SFL self-interactions are not the only role of RAA in inhibition of HIV infection.

### 3.5. Deletions in the N-Terminus of the RAA Modulate SFL Association with the Ribosome

**Figure 6:**
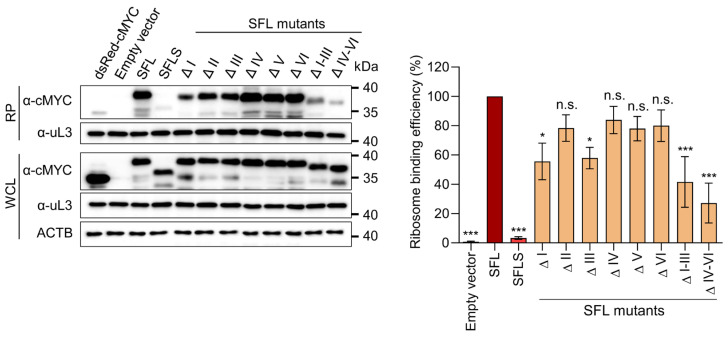
Effect of RAA deletions on ribosome binding. HEK293T cells were transfected with plasmids encoding SFL, SFLS, and the indicated SFL variants equipped with a C-terminal MYC antigenic tag. At 48 h post-transfection, cells were lysed and WCL was loaded on a sucrose cushion and centrifuged for 60 min at 100,000 rpm at 4 °C. The ribosomal pellet (RP) and WCL were analyzed by immunoblot using anti-MYC antibody. The large ribosomal subunit protein uL3 served as a loading control. A representative blot is shown in the (**left panel**); irrelevant lanes were excised. Up to five replicates were quantified by the software ImageJ, and the cMYC signals in RP and WCL were normalized against the corresponding uL3 signals and the ratio of cMYC signals in RP versus WCL was calculated (**right panel**). The ratio obtained for SFL was set as 100%. The average of four (mutants ΔI, ΔII, ΔIII, ΔIV, ΔV, ΔVI) or five (SFL, SFLS, Δ I–III, Δ IV–VI) independent experiments is shown; error bars indicate standard error of the mean (SEM). One-way analysis of variance (ANOVA) with Dunnett’s multiple comparison test was performed to determine whether differences between cells expressing SFL, SFLS, SFL mutants, or empty vector were statistically significant (not significant [n.s.]; *, *p* ≤ 0.05; ***, *p* ≤ 0.001; *n* = 5). SFL, shiftless; SFLS, shiftless short; WCL, whole cell lysate.

SFLS was previously shown to be unable to associate with ribosomes [4]. This prompted us to study whether the lack of antiviral activity of our deletion mutants was due to lack of ribosome association. To test ribosome binding, cMYC-tagged SFL, SFLS, and SFL mutants were expressed in HEK293T cells, and proteins interacting with ribosomes were detected by immunoblot after isolation of the ribosomal pellet (RP) of the WCL using sucrose cushion ultracentrifugation. No SFL sedimentation was observed in the absence of ribosomes, excluding the possibility that large multimers of SFL were co-sedimenting with ribosomes instead of binding to them (Figure S1A). In addition, the interactions between SFL and SFL mutants with ribosomes were disrupted under high salt conditions, indicating that these interactions represent bona fide ribosome binding (Figure S1B). Analysis of ribosomal pellets showed that SFL, but not SFLS, was able to associate with ribosomes (Figure 6), in agreement with previously published data [4]. SFL mutants associated with ribosomes, albeit to a different extent, as mutants with large deletions (ΔI–III, ΔIV–VI) and some N-terminal deletions (ΔI, ΔIII) in the RAA region showed a somewhat lower ribosome association efficiency (Figure 6). The expression levels of all mutants and the amounts of ribosomes in all ribosome pellets and in the WCL samples were comparable (Figure 6). Thus, our results confirm that the RAA region is required for interactions with the ribosome and, together with the findings discussed above, reveal that deletions in the C-terminal portion of the RAA are compatible with SFL oligomerization and ribosome association but not antiviral activity.

### 3.6. Deletions in the RAA Affect Binding to the FSE Region of the HIV mRNA

SFL was suggested to interact with the HIV-1 Gag-Pol mRNA [4,20]. To examine RNA binding of the SFL mutants, cMYC-tagged SFL proteins, SFLS, or untagged GFP as a control were expressed in HEK293T cells and cell lysates were incubated with a fluorescence-labeled mRNA containing the HIV-1 FSE. SFL–RNA complexes were immunoprecipitated by Dynabeads-coupled cMYC antibodies and the pulled down SFL–RNA complexes were analyzed on urea PAGE. The signal observed with the GFP sample was considered background, because the untagged GFP does not bind to cMyc-antibodies. 

**Figure 7:**
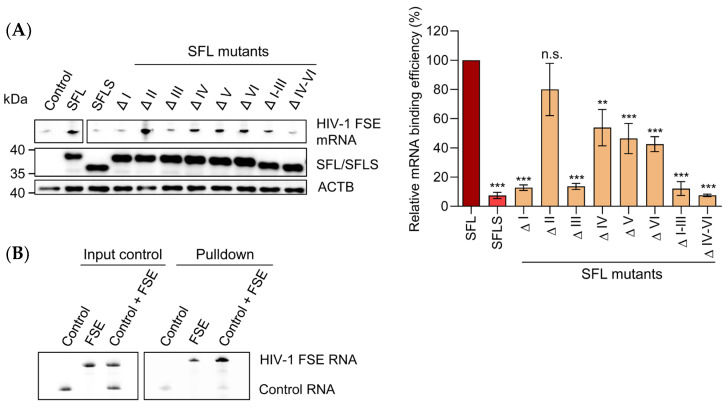
Deletions in the N-terminus of the RAA abrogate SFL binding to the HIV FSE. (**A**) HEK293T cells were transfected with plasmids encoding SFL, SFLS, or the indicated SFL mutants equipped with a C-terminal MYC-tag. A plasmid encoding GFP served as negative control. At 48 h post-transfection, the cells were lysed, fluorescence labeled HIV-1 FSE mRNA was added to cell lysates, and proteins were precipitated with anti-MYC antibody. Co-precipitated HIV-1 FSE mRNA was loaded on a urea gel and the subsequent fluorescence signal was quantified. Expression of SFL, SFLS, and SFLS mutants in WCL was confirmed via immunoblot using anti-MYC antibody. β-Actin (ACTB) expression in WCL served as a loading control. A single representative blot is shown in the left panel; irrelevant lanes were excised. Three biological replicates were quantified by the software ImageJ (right panel). SFL, SFLS, or SFL mutants were normalized to ACTB and in turn used for normalization of HIV-1 FSE mRNA signals. RNA signal measured upon precipitation of SFL was set as 100%. The average of three independent experiments is shown; error bars indicate the standard error of the mean (SEM). One-way analysis of variance (ANOVA) with Dunnett’s multiple comparison test was performed to determine whether differences between cells expressing SFL and SFLS or SFL mutants were statistically significant (not significant [n.s.]; **, *p* ≤ 0.01; ***, *p* ≤ 0.001; *n* = 3). (**B**) HEK293T cells were transfected with plasmid encoding SFL with a C-terminal MYC antigenic tag and WCL were prepared. Subsequently, WCL were incubated with an equimolar mixture of fluorescently tagged HIV-1 FSE mRNA and a control mRNA containing multiple secondary structures. Competitive protein–RNA binding was analyzed via urea–PAGE as described above (right panel). Input control mRNA, HIV-1 FSE mRNA, and a mixture of both are shown in the left panel. The results of a representative experiment are presented and were confirmed in two independent experiments. SFL, shiftless wild type; SFLS, shiftless short; FSE, frameshifting stimulation element; WCL, whole cell lysate.

Our results confirmed that SFL interacts with the HIV-1 mRNA FSE, whereas SFLS lacked RNA-binding activity, although both proteins were expressed to a similar extent (Figure 7A). Removal of region I, III, or I–III, as well as deletion of the last 18 amino acids in the RAA, prevented HIV-1 FSE binding to the same extent as the deletion of the entire RAA (SFLS) (Figure 7A). Removal of amino acids 7–12 (ΔII) of the RAA region had no appreciable effect, whereas deletions within the C-terminal portion of the RAA region (ΔIV, ΔV, and ΔVI) resulted in an intermediate phenotype (Figure 7A). Notably, SFL did bind to a control RNA containing random RNA secondary structures but no FSE (Figure S2). To test whether SFL preferentially binds to RNA containing HIV-1 FSE, we performed similar experiments in the presence of both HIV-1 FSE and control RNAs. SFL preferentially bound to RNA containing HIV-1 FSE (Figure 7B). Collectively, our results indicate that an intact RAA region is required for efficient mRNA binding, although it is less important for SFL self-interactions (Figure 5C) and association with the ribosome (Figure 6).

## 4. Discussion

The present study provides evidence that an intact RAA region is required for SFL-mediated suppression of –1PRF in different cell types and antiviral activity in the context of HIV-1 infection. Furthermore, we identified RAA mutants that failed to bind the HIV FSE-containing mRNA and to suppress production of infectious HIV particles, although these mutants were able to self-associate and bind to the ribosome. Overall, these results support the concept that RAA is essential for inhibition of –1PRF and provide tools for further mechanistic studies on SFL antiviral activity.

SFL was identified as an IFN-induced antiviral factor in the context of DENV infection where it blocks expression of viral genes by interacting with the viral mRNAs and cellular RNA-binding proteins, such as the polyA-binding protein C1 (PABPC1) [16]. During translation initiation, PABPC1 interacts with eIF4G, a subunit of the 5′ cap-binding eIF4E complex [39], and SFL binding to this complex might disturb translation initiation. A separate study reported that SFL localizes to DENV replication complexes via binding to HuR/ELAVL1 [40], an RNA binding protein that is involved in RNA decay pathways [41]. Recombinant SFL also interacts with RNAs from a variety of viruses such as infectious bronchitis virus (IBV), encephalomyocarditis virus (EMCV), and severe acute respiratory syndrome coronavirus 1 (SARS-CoV-1) and 2 (SARS-CoV-2) [15], and reduces the efficiency of –1PRF [20]. Moreover, SFL inhibits –1PRF in numerous retroviruses, including human T cell leukemia virus type II (HTLV-2) [34], mouse mammary tumor virus (MMTV) [42], Rous sarcoma virus (RSV) [43], and simian immunodeficiency virus (SIVmac) [13,37]. Thus, SFL exerts broad antiviral activity [13], and RNA interactions might be required for inhibition of –1PRF [20].

In the context of HIV infection, SFL, but not SFLS, interacts with the ribosome and interferes with a –1PRF event that is required for expression of the HIV Gag-Pol polyprotein and for viral infectivity [13]. The present analysis shows that SFL, which exerts antiviral activity, can form oligomers, whereas SFLS, which fails to inhibit HIV infection, does not self-interact. Multimers likely form by RAA binding to a so-far unidentified SFL region, rather than RAA–RAA interactions, considering that SFLS fails to self-interact but still interacts with SFL. Furthermore, most SFL mutants with a tendency to decreased self-interactions also exhibited reduced ribosome interactions, suggesting that these processes might be linked. 

Several deletions in the RAA that reduced SFL binding to the HIV FSE, as well as its antiviral activity, had a less-pronounced effect on SFL self-interactions and ribosome association (mutants ΔI, ΔIII, ΔI–III, and ΔIV–VI). These findings point towards an essential role of RNA binding in SFL antiviral activity, in keeping with strong RNA binding being required for efficient suppression of –1PRF [20], and the respective mutants might be valuable tools for further analysis of the antiviral mechanism underlying suppression of –1PRF and antiviral activity. However, it is also noteworthy that deletions in the C-terminal portion of the RAA (mutants ΔIV, ΔV, and ΔVI) abrogated inhibition of –1PRF and antiviral activity but did not reduce self-interactions and diminished ribosome association and RNA binding by less than 50%. This may indicate that the RAA-mediated SFL self-interactions are not sufficient for inhibition of HIV infection. Alternatively, the antiviral activity of SFL may depend on the size or structure of multimers, which can be altered by the mutations in the C-terminal part of RAA, but which are not monitored by the methods that we apply here. Moreover, the moderate reduction in ribosome association and RNA binding might have been sufficient for loss of antiviral activity in keeping with strong RNA binding being required for efficient suppression of PRF [20]. Finally, one might speculate that SFL self-interactions as well as ribosome and FSE interactions might be necessary but not sufficient for antiviral activity. In this scenario, the RAA region would be required for a so-far unknown biological activity of SFL that is essential for suppression of –1PRF, a possibility that deserves further investigation.

Some limitations of our study should be noted. For one, SFL moderately interfered with reporter expression that was not dependent on –1PRF (Figure S3). The reason for this interference and the specificity of this effect are at present unclear. However, it is noteworthy that a whole cell proteomic analysis revealed that stable expression of SFL in Huh-7 cells inhibited HCV infection but did not induce changes in the expression levels of cellular proteins [19]. Thus, the modest –1PRF-independent suppression of reporter expression in our assay might be unspecific and caused by protein overexpression upon transient transfection. We also noted that the effects of deletions in the RAA on inhibition of –1PRF largely, but not completely, mirrored the effects on Gag-Pol expression. Although we cannot exclude that these differences are biologically meaningful, we feel that they rather reflect the limited dynamic range of our assays. Further, the effects of the deletions studied here may be direct, i.e., deletions in the RAA region might destroy protein and/or RNA binding motifs, or indirect, i.e., deletions may impact protein folding. For instance, deletions in the RAA region that reduce binding of HIV RNA might alter the conformation of the adjacent zinc finger, which has been implicated in binding to viral RNAs [20]. Finally, it should be noted that although various cell lines were examined for suppression of –1PRF by SFL, lymphocytes, the primary targets for HIV infection, remain to be examined.

Collectively, our study highlights the SFL RAA region as a modulator of SFL self-interactions, SFL interactions with the ribosome and the frameshifting RNA, and SFL-mediated suppression of –1PRF. Structural and further mutagenic analyses will be required to elucidate why the RAA is a master regulator of SFL antiviral activity. 

## Data Availability

The data presented in this study are available on request from the corresponding author.

## Supplementary Materials

The following supporting information can be downloaded at: https://www.mdpi.com/article/10.3390/v14071454/s1, Figure S1: SFL multimers do not co-sediment with ribosomes and ribosomal interaction of SFL can be eliminated by high salt washes; Figure S2: SFL interacts with a control RNA containing random RNA secondary structures but no FSE; Figure S3: Moderate unspecific inhibition of reporter expression by SFL.
