# Supplementary material for: Mutagenic Analysis of the HIV Restriction Factor Shiftless
Source: Viruses. 2022 Jun 30;14(7):1454. doi: 10.3390/v14071454 (PMC9324250; doi:10.3390/v14071454)
Supplement: Supplementary file 1 [file viruses-14-01454-s001.zip › viruses-1746381-supplementary.pdf]

Supplemental Figure S1

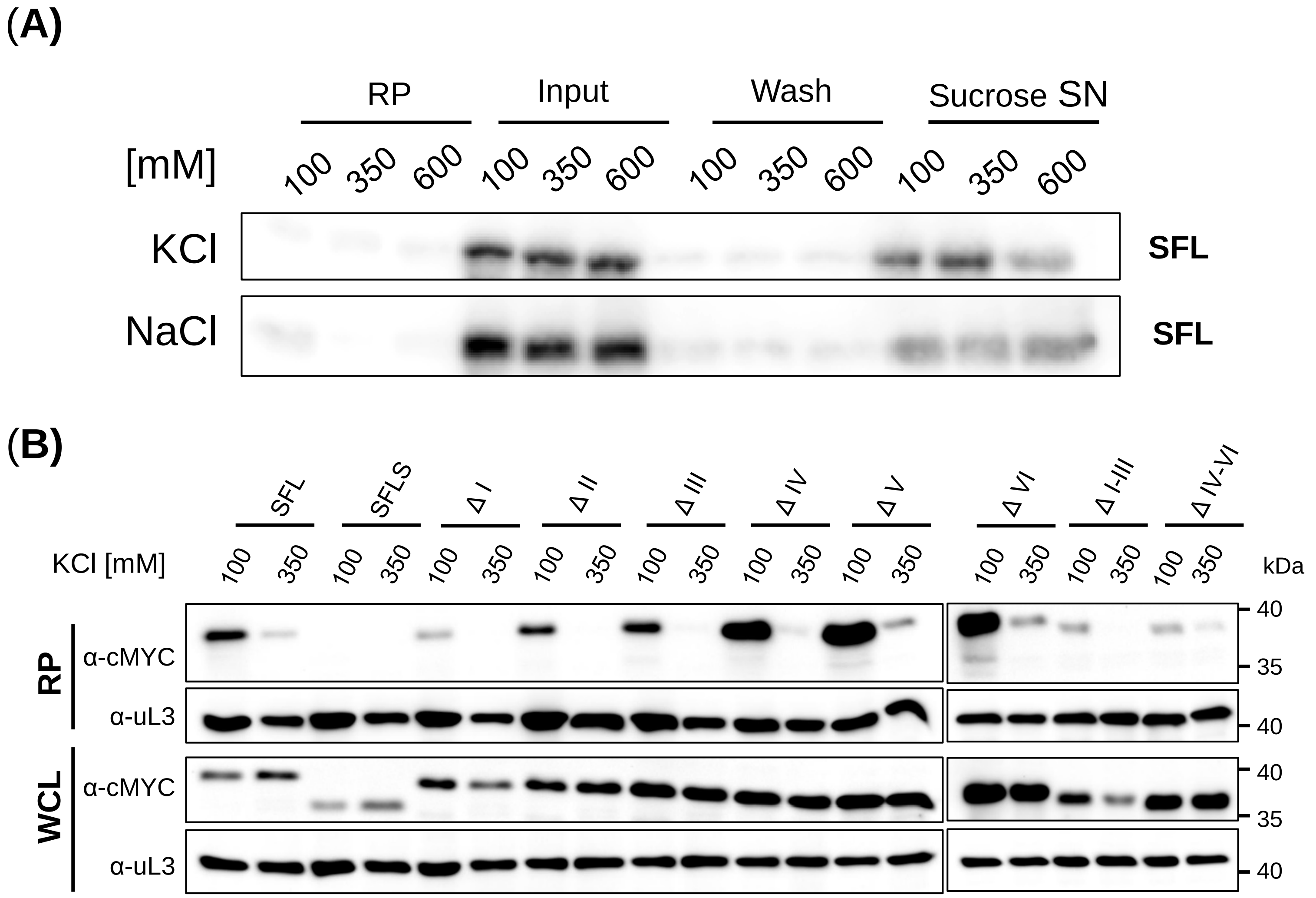

Figure S1. SFL multimers do not co-sediment with ribosomes and ribosomal interaction of SFL can be eliminated by high salt washes. **(A)** Purified SFL treated with KCl or NaCl at the indicated concentrations was loaded on a sucrose cushion and centrifuged for 60 min at 100,000 rpm at 4°C. Pellets were washed with buffers containing the indicated salt concentrations, washes and sucrose cushion supernatants (SN) were analyzed by immunoblot using anti-SFL antibody. Purified SFL not subjected to centrifugation served as positive control (input). One out of two blots is shown and irrelevant lanes were excised. **(B)** HEK293T cells were transfected with plasmids encoding SFL, SFLS and the indicated SFL variants equipped with a C-terminal MYC antigenic tag. At 48 h post-transfection cells were lysed and WCL was loaded on a sucrose cushion and centrifuged for 60 min at 100,000 rpm at 4°C. The ribosomal pellet (RP) was washed either with 100 mM or with 350 mM KCl. RP and WCL were analyzed by immunoblot using anti-MYC antibody. The large ribosomal subunit protein uL3 served as a loading control. A representative blot is shown, irrelevant lanes were excised. Similar results were obtained in two separate experiments. SFL, shiftless; SFLS, shiftless short; RP, ribosomal pellet; WCL, whole cell lysate; KCl, potassium chloride; NaCl, sodium chloride

Supplemental Figure S2

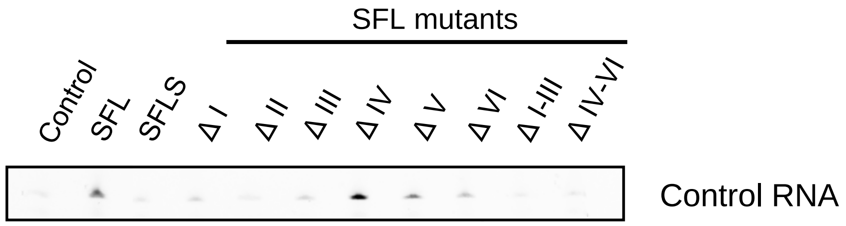

Figure S2. SFL interacts with a control RNA containing random RNA secondary structures but no FSE. HEK293T cells were transfected with plasmids encoding SFL, SFLS or the indicated SFL mutants equipped with a C-terminal MYC-tag. A plasmid encoding GFP served as negative control. At 48 h post-transfection the cells were lysed, fluorescently labeled control mRNA, which lacks FSE but not mRNA-typical secondary structures, was added to cell lysates and proteins were precipitated with anti-MYC antibody. Co-precipitated control mRNA was loaded on a urea gel and the subsequent fluorescence signal was detected via urea PAGE. A representative immunoblot is shown from which irrelevant lanes were excised. Similar results were obtained in two separate experimentsSFL, shiftless; SFLS, shiftless short

Supplemental Figure S3

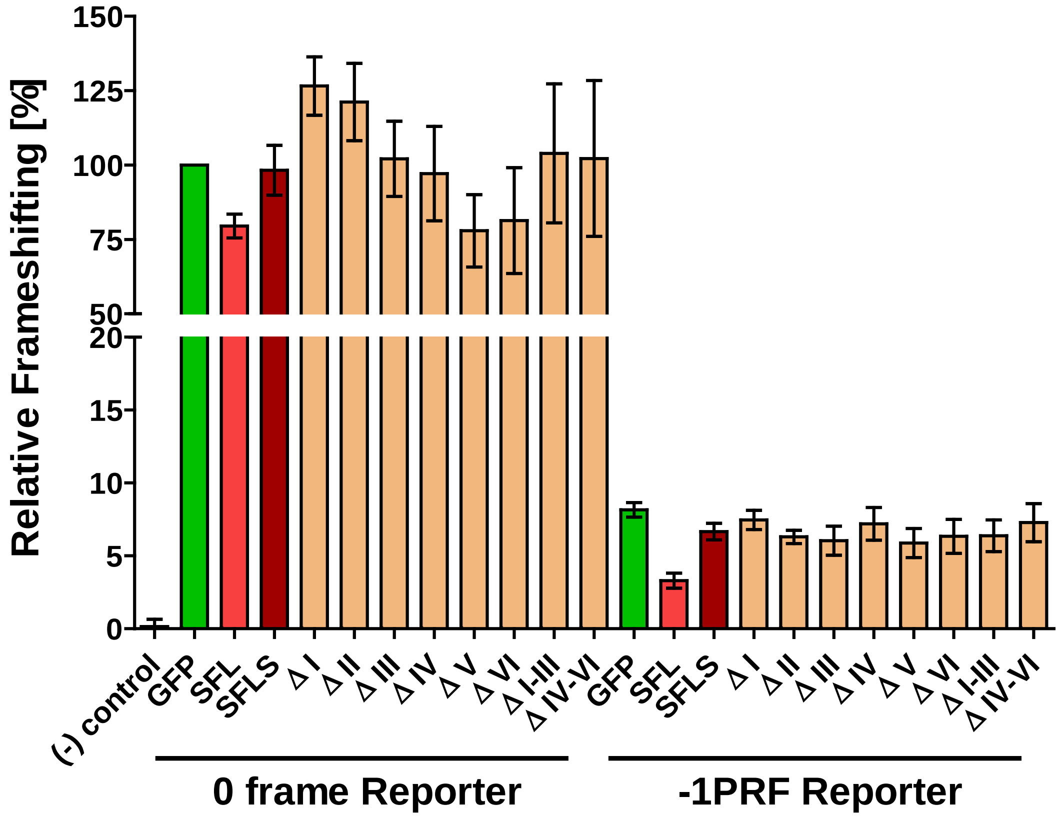

Figure S3. Moderate unspecific inhibition of reporter expression by SFL. A549 cells were transfected with plasmids encoding SFL, SFLS or SFL mutants jointly with the –1PRF reporter plasmid (–1PRF reporter) or the reporter plasmid with a mutated slippery site and RLuc in frame with Flu (0 frame reporter). As negative control, a reporter construct was used with a mutated slippery site and the downstream Fluc in -1 open reading frame (-Control). To determine expression of luciferases in the absence of SFL and SFL variants, a GFP expression plasmid was co-transfected. Luciferase signals in cell lysates were measured at 48 h post-transfection. Signals measured for the 0 frame reporter co-transfected with the GFP plasmid were set to 100%. The average of five biological replicates conducted with technical triplicates is shown, error bars indicate SEM. SFL, shiftless; SFLS, shiftless short
